# Microbiological and 16S rRNA analysis of sulphite-reducing clostridia from river sediments in central Italy

**DOI:** 10.1186/1471-2180-8-171

**Published:** 2008-10-08

**Authors:** Stefania Marcheggiani, Marcello Iaconelli, Annamaria D'angelo, Elio Pierdominici, Giuseppina La Rosa, Michele Muscillo, Michele Equestre, Laura Mancini

**Affiliations:** 1 Department of Environment and Primary Prevention, Istituto Superiore di Sanità, Viale Regina Elena 299, 00169 Rome Italy; 2 Department of Cell Biology and Neurosciences, Istituto Superiore di Sanità, Viale Regina Elena 299, 00169 Rome Italy

## Abstract

**Background:**

Microbiological indicators are commonly used in the assessment of public health risks associated with fecal contamination of freshwater ecosystems. Sediments are a reservoir of microorganisms, and can thus provide information on past pollution events, not obtainable through the testing of surface water. Moreover, pathogens present in sediment may represent future threats to human health. *Clostridium perfringens*, a typical colonizer of sediments, has been suggested as an alternative indicator of fecal pollution. In order to be suitable for such purpose, the microorganism should be widely distributed in contaminated environments. The objective of this study was thus to determine the composition of the anaerobic community in sediment samples of the lower Tiber basin, in central Italy, through a combined approach involving granulometric analysis of sediment samples, as well as a microbiological and molecular (16S rRNA) analysis of strains.

**Results:**

Granulometry showed a similar, clayey sediment composition, in most sampling sites. The microbiological method, employing, an adaptation of the standard method, proved to be effective in isolating anaerobic bacteria from the environmental matrix for the purpose of genetic analysis. Eighty-three strains of bacteria were isolated and the partial 16S rRNA gene sequenced. While biochemical analysis detected only *C. perfringens *strains, phylogenetic analysis indicated the presence of three clusters: *C. perfringens, C. bifermentans *and *B. cereus*, comprising eight taxa. *C. perfringens*, the commonest in almost all sediment sampling sites, was present in all sites, and in both seasons (seasonal sampling was carried out only along the Tiber and Aniene rivers). None of the described genetic profiles showed complete similarity with GenBank sequences.

**Conclusion:**

The study underlines the value of *C. perfringens *as an alternative microbial indicator of fecal contamination in river sediments. This is supported by the bacterium's presence in all sampling sites, and in both seasons, coupled with its detectability using commercial diagnostic kits.

The study also illustrates the presence of an anaerobic community of considerable biodiversity in the lower Tiber basin, with *C. perfringens *as its main component. The 16S rRNA analysis, while confirming the phylogenetic relationships among isolated species, also showed haplotype patterns different from those present in the NCBI database.

## Background

Wastewater effluents play an important role as sources of fecal contamination in freshwater environments. These nonpoint sources of fecal pollution are broadly distributed in both urban and agricultural areas. Standard-based water quality assessment is an essential component of monitoring programs for the protection of human health. Microbiological indicators such as total coliforms, fecal coliforms and fecal streptococci are widely used both in monitoring programs of this kind [1,2] and in human health risk assessment [3,4]. These indicators may also point to the presence of other non-bacterial pathogens such as enteric viruses and parasitic protozoans [5,6]. Several studies have highlighted the value of sediments as indicators of pollution. Indeed, sediments – as potential reservoirs for bacteria and viruses in aquatic environments – are able to provide information on past pollution events no longer detectable in water samples, as well as on the presence of pathogens, which may in some cases pose a future threat to human health.

The higher occurrence of microbial indicators of fecal contamination in sediments as compared to other aquatic environments has been attributed to the sorption of microorganisms onto particles suspended in water, which subsequently settle to the bottom [7-10] and accumulate. Various authors observed higher microbial survival rates in sediment as compared to other aquatic environments, possibly due to the absence of abiotic (UV radiation, salinity, toxic agents) and biotic factors (predation, parasitism, low nutrient availability), which, in surface water, act synergistically to inhibit bacterial growth [11-17]. Others showed higher rates of microbial activity in the sediment surface layer, which is commonly rich in organic matter and thus able to provide suitable habitats for different strains of anaerobes [18,19].

The occurrence of anaerobic microorganisms in freshwater environments is generally linked to the input of sewage outfalls. Some of these organisms are able to survive adverse environmental conditions by forming resting cells (spores) [20,21]. *Clostridium perfringens*, a Gram-positive anaerobic spore-forming bacterium of the genus *Clostridium*, has been suggested [22-28] and successfully used as an alternative indicator of fecal contamination in aquatic environments due to its wide distribution in nature and to its adaptation to a variety of habitats such as soils, sediments and sewages. Moreover, this organism has been found in air, dust, water, and even food [29,30]. Spores produced by this organism are very resistant to disinfection and their presence in filter units indicate treatment inefficiencies [31].

Studies of *C. perfringens *in freshwater ecosystems have been fewer than those performed on marine environments. *Robles et al*. confirmed the value of *C. perfringens *as a conservative indicator of fecal contamination from sewage disposal in high-mountain lakes [32]. Sorensen *et al*. were able to detect traces of unfiltered sewage in small streams by comparing the concentrations of *C. perfringens *spores downstream from wastewater treatment plants [33].

Although the World Health Organization recommends *C. perfringens *as a useful indicator of fecal pollution in water quality surveys [34], this microorganism has been adopted in Europe exclusively as an additional source of water quality information [35-37].

The sequence of the 16S rRNA gene has been widely used as a phylogenetic marker to study genetic relationships between different strains of bacteria (phylogeny). The analysis of this gene can therefore be considered a standard method for the identification of bacteria at the family, genus and species levels [38-44], and has in fact been included in the latest edition of *Bergey's Manual of Systematic Bacteriology *[45].

The objective of this study was to determine the composition of the anaerobic community in sediment samples of the lower Tiber catchment area, in central Italy, through a combined approach involving granulometric analysis of sediment samples, followed by microbiological and molecular (16S rRNA) analyses of bacterial strains isolated from these samples.

## Results

### Sediments and microbiological analysis

With the exception of samples collected from one of the tributaries, the Farfa river, the granulometric composition of all samples was mostly clayey (Table 1).

**Table 1:** Sediment composition in the thirteen sampling sites studied

**Sampling site CODE**	**Clay (%)**	**Silt (%)**	**Sand (%)**
A	70	30	
B	70	30	
C	80	20	
D	70	30	
E	70	30	
F 1	60	20	20
F 2	10		90
F 3	60	40	
F 4	60	40	
T 1	80	20	
T 2	80	20	
T 3	80	20	
T 4	80	20	

Clay, <0.002 mm; silt, 0.02-0.002 mm; sand, >0.02 mm.

The microbiological method used in this study has allowed us to isolate 83 strains of anaerobic bacteria from the sediment samples. To ensure successful growth of colonies, each sample was tested in three suspension aliquots. Replicate sets yielded similar bacterial colony counts (*CFU *per gram of dry weight). The biochemical test was able to detect isolated strains of *C*. *perfringens*, but not other species within the genus.

### Analysis of 16S rRNA fragments

**Figure 1:**
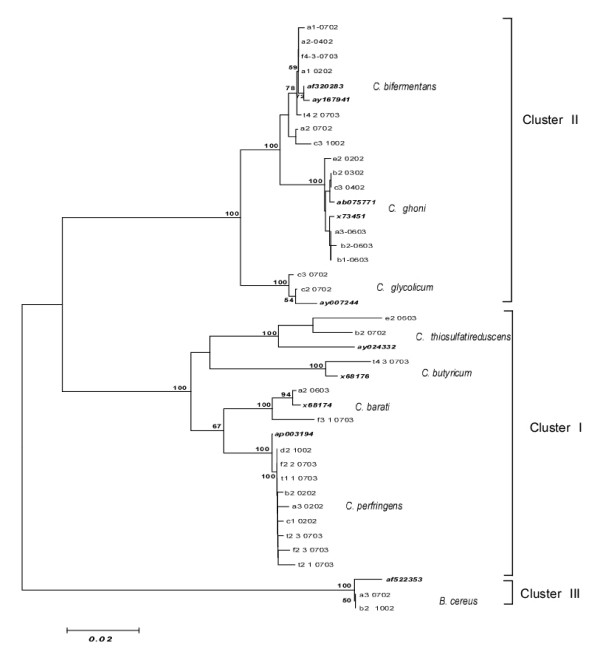
**Phylogenetic tree constructed with the Hasegawa-Kishino-Yano (1985) method, for a 720 bp fragment of the 16S rRNA coding region.** The tree shows the genetic relationships between haplotypes of spore-forming bacterial strains isolated from sediments of the lower Tiber basin. Numbers above branches show bootstrap values expressed as percentages of 100 replications. Database accession numbers are given in bold. Bar, 0.02% sequence divergence.

Eighty three PCR products derived from Gram-positive sulphite-reducing bacteria collected in this study were aligned and matched against NCBI database sequences for the closest phylogenetic neighbors. The resulting phylogenetic tree, based on the neighbor-joining method, is given in Fig. 1. Thirty one haplotypes were observed (Table 2), with a total of 202 variable sites, of which 180 were informative. The transition/transversion (ti/tv) ratio was 1.1. Base frequencies were as follows: A = 0.261, C = 0.216, G = 0.312 and T = 0.211. The higher frequency of G corresponds to what is generally observed among spore-forming bacteria.

**Table 2:** Mean genetic distances (HKY85) between the eight taxonomic units isolated from sediments of the lower Tiber basin

**Species**	**1**	**2**	**3**	**4**	**5**	**6**	**7**	**8**
***C. perfringens***	0.004							
***C. barati***	0.045	0.007						
***C. butyricum***	0.087	0.077	0.009					
***C. thiosulfatireducens***	0.117	0.110	0.118	0.033				
***C. glycolicum***	0.226	0.226	0.281	0.279	0.005			
***C. bifermentans***	0.204	0.213	0.250	0.259	0.044	0.004		
***C. ghoni***	0.212	0.239	0.307	0.273	0.054	0.022	0.006	
***B. cereus***	0.366	0.372	0.392	0.414	0.290	0.294	0.313	0.004

**Figure 2:**
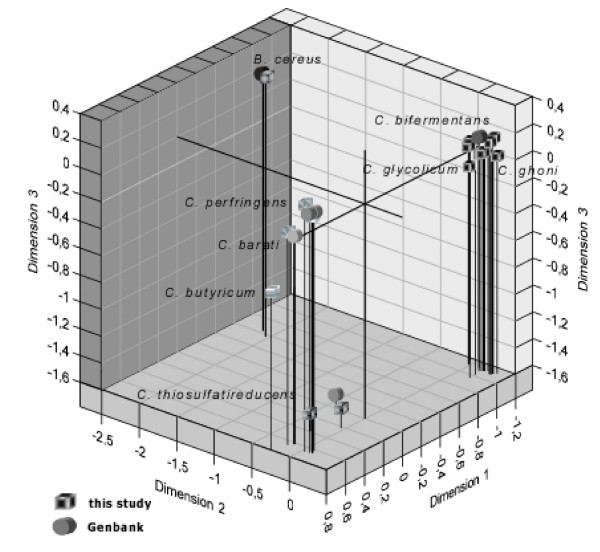
**Multidimensional scaling ordination in three dimensions, based on Kruskal's method. **The plot shows genetic relationships between anaerobic strains isolated from sediments of the lower Tiber basin. Genetic distances between the three clusters are clearly visible, as well as the incomplete homology between the environmental strains isolated (squares), and those previously published in GenBank (circles).

All phylogenetic analyses clearly assigned the bacteria to one of three main clusters of anaerobic bacteria: *C. perfringens *(I), *C*. *bifermentans *(II) and *B. cereus *(III), grouped into two genuses (*Clostridium *and *Bacillus*). Cluster I included the taxa *C*. *perfringens*, *C*. *barati, C*. *thiosulfatireducens *and *C. butyricum*, and cluster II included *C*. *bifermentans*, *C*. *glycolicum *and *C*. *ghoni*. The only member of cluster III was *B. cereus*, with a haplotype presenting a 26-bp insertion at position 78 in our alignment set. A Multidimensional Scaling (MDS) [46,47] plot based on the HKY (Hasegawa Kishino Yano) model [48] revealed a marked heterogeneity within the first cluster, along the third dimension (Fig. 2). The haplotype corresponding to *C*. *thiosulfatireducens *appeared to be the most distant within its cluster, with a genetic distance value of 0.117. The haplotype corresponding to *C. barati *appeared to be the closest to *C*. *perfringens*, with a distance value of 0.045. Genetic distances between the members of the second cluster ranged from 0.04 to 0.05. As for the third cluster, *B*. *cereus *diverged from the other groups, with distance values between 0.39 and 0.29. Mean distance values between the main groups are given in Table 3.

**Table 3:** Haplotypes observed in this study, as identified through sequencing

**Species**	**Sequence Length**^**1**^	**Origin**	**GenBank Accession**
*C. bifermentans*	673	ENV	EF153846
*C. bifermentans*	674	ENV	EF153847
*C. bifermentans*	673	ENV	EF153848
*C. bifermentans*	673	ENV	EF153853
*C. bifermentans*	673	ENV	EF153859
*C. bifermentans*	673	ENV	EF153860
*C. bifermentans*	673	ENV	EF153861
*C. ghoni*	672	ENV	EF153862
*C. ghoni*	677	ENV	EF153864
*C. ghoni*	673	ENV	EF153865
*C. ghoni*	674	ENV	EF153866
*C. ghoni*	673	ENV	EF153867
*C. ghoni*	673	ENV	EF153868
*C. glycolicum*	673	ENV	EF153869
*C. glycolicum*	673	ENV	EF153870
*C. perfringens*	673	ENV	EF153871
*C. perfringens*	672	ENV	EF153873
*C. perfringens*	672	ENV	EF153911
*C. perfringens*	672	ENV	EF153912
*C. perfringens*	672	ENV	EF153913
*C. perfringens*	672	ENV	EF153915
*C. perfringens*	672	ENV	EF153916
*C. perfringens*	672	ENV	EF153917
*C. perfringens*	672	ENV	EF153918
*C. barati*	672	ENV	EF153919
*C. barati*	672	ENV	EF153921
*C. thiosulfatireducens*	672	ENV	EF153922
*C. thiosulfatireducens*	672	ENV	EF153925
*C. butyricum*	676	ENV	EF153926
*B. cereus*	701	ENV	EF153927
*B. cereus*	699	ENV	EF153928

^1 ^Length of sequence used in BLAST search

None of the genetic profiles described in this study showed complete similarity with those previously published in the NCBI database, with similarity scores ranging between 0.95 and 0.99.

**Figure 3:**
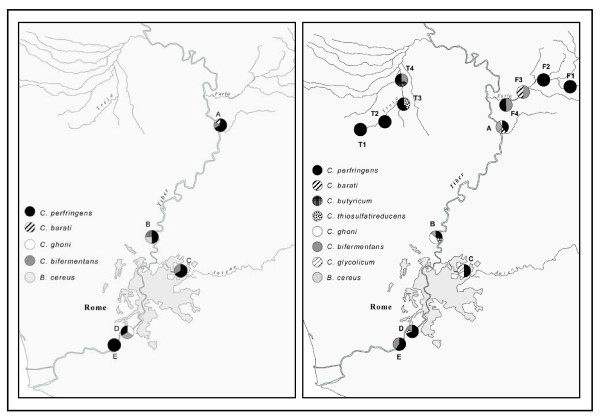
Geographical distribution and frequencies of the genetic profiles observed in different seasons (left figure = cold season; right figure = warm season).

The geographical and frequency distribution of the eight taxonomic units identified is given in Fig. 3. *C*. *perfringens *was found to be the most prevalent in all but one of the sampling sites, accounting for between 33% and 100% of anaerobic beacteria identified. As expected, more biodiversity was observed downstream than upstream, both along the stem of the Tiber and along its tributaries. The nucleotide sequence data reported in this study have been deposited in the NCBI (GenBank) database [accession numbers EF153846 to EF153928].

## Discussion

The rate of pollution of the Tiber river increases considerably downstream, with the growing exploitation of its waters for agricultural, urban and industrial activities. The quality of the river's water decreases remarkably after its confluence with its main tributary, the Aniene river, in the city of Rome. Despite several measures taken in order to reduce the impact of human activities on the Tiber ecosystem, illegal sewage discharge persists in a number of locations, attesting to severe human pressures and water mismanagement. The study of microbial indicators of pollution in sediment, rather than water, could contribute valuable information concerning factors affecting freshwater ecosystems. Sediments may prove useful in this respect, in that they "trap" pollutants and constitute a favourable habitat for various anaerobic bacteria.

Preliminary efforts, involving the application of published protocols [49-51], such as the *Most Probable Number *(MPN) and the *Membrane Filtration *(MF), entailed several problems, due to the growth of atypical colonies, which interfered with the growth of spore-forming microorganisms. To avoid this inconvenience, we implemented an adaptation of the standard protocol, described in the Methods section. As noted above, each sample was tested in three suspension aliquots yielding similar bacterial counts, which attests to the validity of the method employed. This adaptation allowed us to successfully isolate 83 strains of anaerobic bacteria from our sediment samples.

Subsequent genetic analysis assigned the bacteria to one of three clusters: *C. perfringens*, *C*. *bifermentans *and *B. cereus*. The biochemical test, on the other hand, was able to detect isolated strains of *C*. *perfringens*, but not other species within the genus. The low efficiency of the biochemical analysis on the samples collected is probably related to the fact that commercial kits are specifically developed on the basis of the metabolic pathways of clinical strains, and therefore cannot be as selective in discriminating environmental species. It is noteworthy, however, that such kits were able to detect the most frequently present and widely spread bacterium – *C. perfringens*.

The combined application of both microbiological isolation and selection of specific primers for the 16S rRNA gene provided a suitable tool for the detection of the sulphite reducing bacteria present in the layers of sediment of the lower Tiber river basin.

The primers used for PCR amplification are efficient only if, before microbiological analysis, samples are submitted to heat treatment, to inactivate vegetative forms of bacteria. The crucial role of such treatment emerged in a recent, yet unpublished study, in which we found a clear association between the efficiency of the primers and preliminary heat treatment of environmental samples.

Our phylogenetic analysis confirmed the previously described heterogeneity within the genus *Clostridium *[52-54]. The lack of complete similarity between the genetic profiles found in this study and those present in the NCBI database could be attributed to the former being typical of the geographic region of the lower Tiber basin. Additional comparisons with strains originating from other watersheds remain necessary, however, both in order to confirm the exclusivity of the Tiber haplotypes and to evaluate their geographic ranges.

The greater microbial biodiversity observed in samples collected in early autumn seems to be related to seasonal water regimes and temperature fluctuations. Higher temperatures generally promote eutrophication and thus the establishment of anaerobic conditions. The input of sewage is generally enhanced by a higher frequency of rainfall during this season, which is generally followed by unpredictable increments in pollutants that are partly adsorbed by the sediment matrix. The effects of such events on water macrofauna, notably in the form of high fish mortality, are particularly evident following heavy rainfalls. These events can promote the development of suitable habitats for the growth of different species of anaerobes. Further studies should therefore be conducted in order to detect possible relationships between outfall types and the composition of anaerobic microbial communities in river sediments.

The ability of sediment to record former pollution events and the pathogenic nature of sulphite-reducing spore-forming bacteria such as *C. perfringens*, underscore the importance of bacteriological analysis of sediment for the purposes of microbiological risk assessment of inland water ecosystems [55].

## Conclusion

This study underlines the value of *Clostridium perfringens *as a microbial indicator of fecal contamination in river sediments. The presence of this bacterium in all sediment sampling sites, as well as in both seasons (along the main stem of the Tiber, where samples were collected seasonally), combined with the fact that it was the only anaerobic bacterium detectable using commercial biochemical diagnostic kits, lend support to its suitability as an alternative indicator of fecal pollution in water quality surveys.

While further studies are still needed to explore possible relationships between the presence of specific microorganisms in sediments and the effects of such pollution on human health, one may safely assume that sulphite-reducing bacteria – being useful sources of information – are destined to play a key role in the management of freshwater environments. Moreover, information derived from the analysis of sulphite-reducing bacteria in river sediments may prove valuable in water reclamation plans. In this context, the information may be used for purposes such as the evaluation of health risks in a given area, or quality control, to ensure that the health of the water ecosystem in question has in fact been restored or improved.

Although quantitative analysis on the densities of sulphite reducing clostridia in the sediments were not performed in this work, however the qualitative molecular tools employed for the identification of bacteria proved extremely useful not only in confirming the value of *C. perfringens *as a possible bacterial indicator of fecal contamination, but also in allowing the identification of genetic relationships between species. Further quantitative studies will be useful in order to state the suitability of this group of bacteria as indicators. Despite the usefulness of qualitative molecular tools, however, these cannot currently replace classical methods of routine water quality assessment, the latter being quantitatively more informative and easier to execute. An alternative, faster molecular methodology, which may be examined in future studies, is the direct extraction of microbial genome from the matrix and its amplification using selective primers specifically designed for each taxonomic unit.

## Methods

### Geographical area studied and sediment sampling

The Tiber River belongs to one of the largest river systems in Italy, with a catchment area of 17,375 km^2 ^. The river is 405 km long, and runs from the Tuscan-Emilian Apennines to the Tyrrhenian Sea, through four administrative regions. The water volume ranges from 60 m^3^s^-1 ^to 3200 m^3^s^-1 ^with a yearly average of 230 m^3^s^-1 ^[56].

Thirteen sampling sites were selected, four along the main stem of the Tiber, four along the Farfa, four along the Treja and one along the Aniene (Table 4). The aim was to allow a comparison of the microbial community composition in sediments upstream and downstream of the city of Rome Sediment samples were collected seasonally from February 2002 to June 2003 for all sites located along Tiber River and Aniene tributarie while sites along Treja and Farfa were sampled only during the hot season. Sampling was performed using a bucket attached to a winch, that was immersed at a depth of between 1 and 3 meters below the water surface. Samples were transported and stored in sterile 50 ml tubes, at 4 °C [57]. Granulometric characterization was performed on each sediment sample, based on the different sedimentation speeds of silt, clay and sand particles [58,59].

### Isolation of bacteria

Five grams of sediment from each sample were suspended in 45 ml of sterile phosphate buffered saline (PBS) [K_2_HPO_4 _3 g/ml, KH _2_PO_4 _1 g/ml, NaCl 8,5 g/ml; (pH 7,2 ± 0,2)] + 0.1% Tween 80. Three replicates were used for each sample. Suspension samples were then homogenized on a magnetic stirrer (Heidolph, MR 3001) for 30 minutes in order to break up clumps of bacteria. The samples were heat-shocked at 80–85°C for 10 minutes before cultivation, so as to inactivate vegetative bacteria and enhance sporulation. Five ml of the spore-containing suspension was diluted (1:10) with sterile PBS, in 15 ml Falcon vials (BD, USA). For optimal growth, we adopted the following plating technique: 8 ml of SPS (Sulphite Polymixine Sulphadiazine Agar, Oxoid) medium culture was placed in Petri dishes with a 1 ml aliquot of diluted suspension, and incubated in an anaerobic jar equipped with a manometer and a CO_2 _generator, at 36 ± 1°C for up to 24 hours. Black colonies characteristic of anaerobic bacteria appeared.

Colonies were counted directly, and the results expressed as colony-forming units per gram of dry weight of sediment (*CFU*/g_ss_). In order to calculate dry weight, about five grams of sediment were weighted and dried, in triplicate, at 120 ± 1°C for 12 hours. The procedure was repeated until a stable weight was reached.

After counting, colonies were transferred into new plates with TSA (Tryptone Soya Agar, Oxoid) medium and incubated in anaerobic conditions as described above. Single colonies were isolated from each of the three replicates and resuspended in sterile water for DNA extraction. Biochemical identification of strains was then performed using API 20A Strips (Biomerieux, France) according to the manufacturer's instructions.

Positive and negative controls were used to validate the microbiological method. A negative control was generated as follows: after sterilization at 121°C for 15 min, five grams of sediment were suspended into 45 ml of sterile PBS, then plated in medium culture as described above. The same procedure, followed by *in vitro *infection with *C. perfringens *(ATCC 12918), was used to generate a positive control.

Microbiological analyses and genome extractions were performed within 24 hours from sampling; extracted DNA were redissolved in sterile water, aliquoted in small volumes and stored at -80°C until use. All molecular experiments were completed within six months from extraction.

### DNA extraction and 16S ribosomal RNA amplification

One and a half ml of microbial suspension was pelleted by spinning at 6000 **rpm **for 5 min. Extraction of nucleic acids was carried out following the method described in Ausubel *et al*. [60]. The pellets were resuspended in 570 μ1 TE buffer [10 mM Tris-HCl, 1 mM EDTA, (pH 8)] and the suspension mixed with 80 μl CTAB/NaCl solution (10% CTAB in 0.7 M NaCl), 30 μl of 10% SDS and 3 μl of Proteinase K (20 mg μ/ml), and incubated at 55°C for 10 min.

An equal volume of phenol/chloroform/isoamyl alcohol (25:24:1) was added, and the mix was centrifuged at 17000 **rpm **for 5 min. Supernatants were transferred to new tubes, washed with 1 volume of chloroform/isoamyl alcohol (24:1) and spinned at 17000 **rpm **for 5 min. Supernatants were again transferred to new tubes and DNA precipitated at -20°C with two equivalent volumes of 95% ethanol for 30 min. After precipitation pellets were washed with 70% ethanol, dried in a thermoblock at 40°C, and resuspended in 100 μl of 10 mM TE buffer. A 701 bp fragment of the 16S rRNA coding region was selectively amplified with two primers: 728 forward (5'-GGGGAATATTGCACAATGG-3') and 729 reverse (5'-GGGACTTAACCCAACATCTCA-3'). Primers were designed (using Primer3 software) [61], so as to anneal to conserved positions of different *Clostridium *species published on the NCBI database (accession numbers: NC_003366, AF371836, X68174, AF479585, AF458779). In particular the forward primer anneal at position 351–371 and the reverse primer at position 1047–1060 of accession number NC_003366 (*Clostridium perfringens *str. 13, complete genome, GeneID: 988236). Annealing temperature was calculated by the Primer 3 software. The final concentrations of PCR reaction components were: 10 mM PCR Buffer [Tris-HCl (pH 8.8)], 2 mM MgCl_2_, 0.2 mM of each dNTP, 0,2 mM of the two primers, 1 U Taq polymerase (Roche, Mannheim, Germany).

The PCR reaction was performed in a Personal cycler (Biometra T4, Göttingen, Germany), under the following conditions: an initial denaturation step at 94°C for 2 min, followed by 35 cycles at 94°C for 30 s, annealing at 57°C for 30 s and elongation at 72°C for 60 s. The reaction was terminated with a 10-min final elongation step at 72°C. A negative control (sterile deionized water) and a positive control (*C. perfringens *genomic DNA) were included in the experiment to confirm the validity of results.

PCR products (5 μl of each reaction mixture) were visualized by electrophoresis on a 1% agarose gel, in TBE (Tris Borate EDTA) buffer (100 mM Tris-HCl, 90 mM boric acid, 1 mM Na_2_EDTA), with ethidium bromide staining (10 mg/ml), at 90 V for 1 h.

### Partial sequencing of the 16S ribosomal RNA gene

Before sequencing, unincorporated nucleotides and primers were removed from PCR products using the Promega DNA Purification System (Madison, WI, USA), following the manufacturer's protocol. The sequencing reaction was prepared using the CEQ 2000 Dye Terminator Cycle Sequencing with the Quick Start Kit (Beckman Coulter, Fullerton, USA), and was performed in a Personal cycler (Biometra T4, Göttingen, Germany) according to the manufacturer's instructions. Sequences were generated with a Beckman 3000 sequencer (Beckman Coulter, Fullerton, USA) and analyzed using Chromas 2.23 software (Technelysium Pty Ltd, Tewantin, Australia). BLAST searches were performed for each of the sequences obtained, to find its closest phylogentic neighbors.

### Sequences alignment and analysis

The partial sequences of 16S rRNA genes obtained in this study were aligned to each other using ClustalX software, version 1.81 [62,63]. Phylogenetic relationships were inferred by the neighbor-joining method [64] using the phylogenetic inference software Paup*, version 4.0 [65]. The evaluation of the most appropriate model of sequence evolution was computed using ModelTest version 3.0 [66]. For the 16S rRNA gene, this resulted in the selection of the HKY85 + G model. Genetic relationships were also plotted by non-metric multidimensional scaling (MDS) using SYSTAT 9.0 software (SPSS-SCIENCE), which provides an ordering of n variables on a similarity/dissimilarity matrix by means of n points in a three dimensional space [Figure 2].

### Accession numbers of 16S rRNA sequences

The 83 nucleotide sequences reported in this paper have been deposited in the GSDB, DDBJ; EMBL and NCBI nucleotide sequence databases under accession nos. EF153846 to EF153928. Eighty three environmental isolates of *Clostridium*: *C. bifermentans *[GenBank: EF153846 to 153861], *C. ghoni *[GenBank: 153862 to 153868], *C. glycolicum *[GenBank: 153869 to 153870], *C. perfringens *[GenBank: 153871 to 153918], *C. barati *[GenBank: 153919 to 153921], *C. thiosulfatireducens *[GenBank: 153922 to 153925] and *C. butyricum *[GenBank: 153926], and two environmental isolates of *Bacillus cereus *[GenBank: EF153927, EF153928].

## Authors' contributions

SM performed most of the experiments, and participated in the interpretation of data and in the discussion of results. MI designed and performed part of the molecular analysis and was involved in the interpretation of data. AD planned the microbiological analysis. MM carried out the phylogenetic analysis. ME participated as a consultant and hosted SM in his laboratory for DNA sequencing. EP carried out the sampling of sediment. GL critically revised the manuscript. LM initiated the study, designed the experiments, and participated in the discussion of results. SM, MI and LM participated in the writing of the manuscript. All authors read and approved the final manuscript.
